# Most transcription factor binding sites are in a few mosaic classes of the human genome

**DOI:** 10.1186/1471-2164-11-286

**Published:** 2010-05-06

**Authors:** Kenneth J Evans

**Affiliations:** 1 School of Crystallography, Birkbeck College, University of London, Malet Street, London, WC1E 7HX, UK

## Abstract

**Background:**

Many algorithms for finding transcription factor binding sites have concentrated on the characterisation of the binding site itself: and these algorithms lead to a large number of false positive sites. The DNA sequence which does not bind has been modeled only to the extent necessary to complement this formulation.

**Results:**

We find that the human genome may be described by 19 pairs of mosaic classes, each defined by its base frequencies, (or more precisely by the frequencies of doublets), so that typically a run of 10 to 100 bases belongs to the same class. Most experimentally verified binding sites are in the same four pairs of classes. In our sample of seventeen transcription factors — taken from different families of transcription factors — the average proportion of sites in this subset of classes was 75%, with values for individual factors ranging from 48% to 98%. By contrast these same classes contain only 26% of the bases of the genome and only 31% of occurrences of the motifs of these factors — that is places where one might expect the factors to bind. These results are not a consequence of the class composition in promoter regions.

**Conclusions:**

This method of analysis will help to find transcription factor binding sites and assist with the problem of false positives. These results also imply a profound difference between the mosaic classes.

## Background

The DNA sequence has no landmarks to guide the search for transcription factor binding sites: these binding sites may be near the transcription start site but may also be far from it [1,2]. Many papers have examined how these sites might be found computationally [3]. Some methods use a comparison between orthologous regions of different species [4], often treating the problem as one of multiple alignment [5,6]. Other algorithms use a collection of subsequences containing a binding site (for example the promoter regions of coregulated genes or subsequences derived from ChIp-chip experiments) to deduce the form or motif of the binding site which is then used to identify sites in other sequences  — reviews of these methods are given in [7,8]. These methods include Weeder [9], MEME [10], ANN-SPEC [11], MORPH [12] and GLAM [13]. Some authors have proposed a statistical test to decide whether a region of DNA is a regulatory region: two methods [14,15] tested on fly data have been motivated by the hypothesis that the local region around the binding site should be similar to the motif itself. Interestingly, such a tendency would not explain the results of this paper. A distantly related line of research is the modeling of nucleosome positions with the expectation that transcription factor binding sites avoid these positions [16-18]. A number of projects have combined data of several types to predict binding sites: for example [19-21].

The motif-finding methods give the immediate context for the current work. These methods commonly find a large number of false positive binding sites in new sequences [22-24]. As well as a model for the binding site, these methods need a model of the non-binding sequence. The complexity of this model ranges from using single nucleotide frequencies (the default for MEME [10]), to modeling the background as a number of states [25]. Using a Hidden Markov Model, that study found that a useful level of complexity was four states with the probability of a base at a given position depending on the state and the previous base. It is convenient to refer to these states as "mosaic classes" because they are short  — about 50-100 bases long. However, the emphasis has been on using no more complexity than is needed to assist the motif finding: there appears to have been little work to find the best model for the bulk DNA and this paper addresses this problem. It is plausible that such an analysis will be useful because much of the genome gets its character from local evolutionary processes [26-28] which would be well modeled by these kinds of classes. Short repetitive elements would also be well described.

In this paper, we draw a distinction between occurrences of motifs in the DNA sequence which are sites where a transcription factor might bind (and do bind in *in vitro *experiments [29]), and binding sites where factors are experimentally found to bind *in vivo*. There is also the difference between binding sites and the subset which are proven to affect transcription [30], but this point is not considered in this paper.

We find that the DNA sequence may be described in terms of short subsequences: each subsequence belonging to one of 38 states, or mosaic classes, each with its own distribution of base frequencies. These classes come in pairs because of the equivalence between the strands. For a set of seventeen transcription factors from different families of factors, 75% of actual binding sites are in the same set of four pairs of preferred classes which account for only 26% of the bases of the genome. However, only 31% of the motifs for these factors are in the same classes. This tendency is observed for all seventeen transcription factors. These results are *not *a consequence of the different base composition near transcription start sites.

## Results and discussion

### Classes Found

By analysing sequences taken at random from the whole human genome, we find the pairs of mosaic classes described in Table 1. The parameters of the mathematical model describing these classes are given in Additional File 1. The classes are generally short in the range 10 to 100 bases, but one pair (number 6) has an average length of over 500 bases. As one would expect most classes are poor in CpG doublets, but one pair (number 14) has a CpG doublet proportion of 10%.

**Table 1:** Summary statistics for the mosaic classes of the human genome

Class pair	A	C	G	T	Length	CpG proportion	Proportion of genome
1	2	3	4	5	6	7	8
1	0.169	0.234	0.104	0.492	5.4	0.006	0.143
2	0.230	0.220	0.243	0.307	84.7	0.011	0.134
3	0.314	0.139	0.118	0.428	71.5	0.003	0.132
4	0.235	0.281	0.133	0.351	64.9	0.005	0.120
5	0.279	0.175	0.185	0.361	55.3	0.004	0.116
6	0.215	0.165	0.188	0.432	504.7	0.005	0.092
7	0.183	0.336	0.205	0.276	55.6	0.009	0.069
8	0.177	0.392	0.194	0.237	33.6	0.029	0.044
9	0.167	0.379	0.236	0.218	48.2	0.027	0.042
10	0.145	0.242	0.309	0.303	18.2	0.017	0.022
11	0.199	0.453	0.240	0.108	14.4	0.056	0.017
12	0.175	0.021	0.079	0.725	14.4	0.000	0.014
13	0.252	0.359	0.126	0.263	10.5	0.003	0.012
14	0.125	0.454	0.252	0.169	25.8	0.096	0.009
15	0.217	0.357	0.124	0.301	71.4	0.010	0.008
16	0.223	0.045	0.261	0.471	14.0	0.007	0.007
17	0.003	0.066	0.006	0.925	11.9	0.000	0.006
18	0.099	0.402	0.071	0.429	5.1	0.024	0.006
19	0.021	0.434	0.023	0.522	33.5	0.003	0.005

Each row refers to two classes, which we refer to as the *a *and *b *classes, each with the reverse complement properties of the other. The *a *class is the one with the higher percentage of T+C, and the base frequencies for this class are shown in columns 2-3-4-5. The frequencies for the *b *class are the same but with the A/T and C/G frequencies interchanged. Column 6 gives the mean length of the class in bases and column 7 gives the proportion of doublets within the class that are CpG: these two quantities are the same for both the *a *and *b *classes. Column 8 gives the proportion of bases in the genome within both classes of the class pair: the total of column 8 is therefore 1.0. The class pairs have been numbered by the proportion of bases they contain. All values have been calculated from the fitted HMM: the parameters of this model are given in Additional File 1.

**Figure 1:**
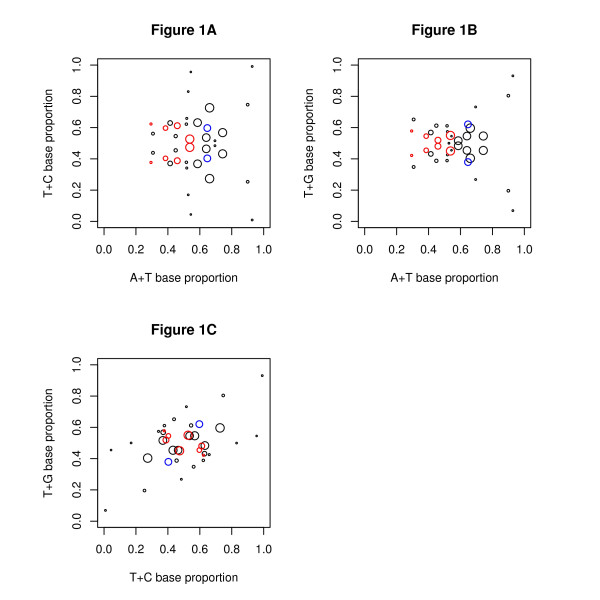
**The mosaic classes of the human genome**. Each circle represents one of the mosaic classes. The position of the circle shows the A/C/G/T content:-(a) T+C by A+T, (b) T+G by A+T, (c) T+G by T+C. The area of the circle shows the proportion of the genome contained in the class. The preferred classes (pairs 2, 7, 9 and 14) contain the most transcription factor binding sites and are shown in red. The classes of pair 6 are comparatively long: they are plotted in the same way as the others but are shown in blue. The purpose of this plot is to give a visualisation of the mosaic classes. The genome is AT rich so it is not surprising that there is a preponderance of classes on the right hand side of Figures 1A and 1B. Strand symmetry gives an exact symmetry about the horizontal line T+C = 0.5 in (a) and T+G = 0.5 in (b). In (c), the strand symmetry shows itself as an axial symmetry--that is the line between paired classes is bisected by the central point. All proportions have been calculated from the steady state of the HMM.

To visualise the mosaic classes, we have plotted them by their A, C, G, T content. To do this we have used the variables T+A, T+C, T+G  —  see Figure 1. These variables have been used because the A, C, G, T proportions form a three dimensional space (one degree of freedom is lost as the proportions sum to one), which is most naturally interpreted as a tetrahedron. Plotting the space using any two of these variables gives an undistorted view of the tetrahedron with the four corners of the tetrahedron at the four corners of the plot. These variables also maintain the symmetry between the bases as the variables T+A, T+C, T+G are one minus the remaining three pairs of variables C+G, A+G, A+C.

It would be possible to use sequences from a defined subset of the human genome to derive the mosaic classes, and such an analysis might give different results. Interesting possibilities include promoter regions, transcribed regions, non-transcribed regions and the genome masked of transposons and/or repetitive elements. Using the whole genome has the advantage of simplicity and ensures that nothing has been left out: it is the obvious baseline analysis and is justified by the results we obtain. Whether a different subset of the genome gives a more biologically relevant set of classes is a matter for research.

### Symmetries

DNA has two strands which are chemically indistinguishable, with the structure found by Watson and Crick of As paired to Ts and Cs to Gs. When the base frequencies of a class are counted, it is necessary to choose one of the strands arbitrarily for the measurement. To discuss the effect of this symmetry, consider a hypothetical class, (called *H*), in which 40% of the bases are A, 20% are C, 20% G, and 20% T. There are logically two contradictory possibilities: a) Such a class as *H *cannot exist — exact strand symmetry applies everywhere including within a class, so that in a class there will be the same number of As as Ts and Cs as Gs. This would imply strong constraints on the base frequencies of any class. b) When the genome is examined — with the strands being equally likely to be used as the measurement strand — it will appear that there is another class *K *whose characteristics are the reverse complement of *H*, that is 40% T, 20% G, 20% C, 20% A. We find from our analyses that statement a) is false: if it were true, then the classes found would occur on the line of symmetry T+C = 50% in Figure 1A. This result is not unexpected because there is a literature [31,32] on A-T and C-G strand symmetry, which suggests that both on the small scale [28,33] and the large scale [34,35] there may a preferred strand.

It is possible for a large scale feature of the genome to be used to break the symmetry between the strands by defining one strand as the measurement strand — for example, in DNA replication one of the strands is the leading strand. However, we expect that classes will still be found in symmetric pairs, even if some class-pairs have a preferential orientation with respect to the defined strand. This remark is based on early analyses concerning transcription — details not shown.

In Figure 1, there are hints of other approximate symmetries, (about a vertical axis in Figure 1A and 1B and about a sloping line from lower left to upper right in Figure 1C).

### Transcription factor binding sites

**Figure 2:**
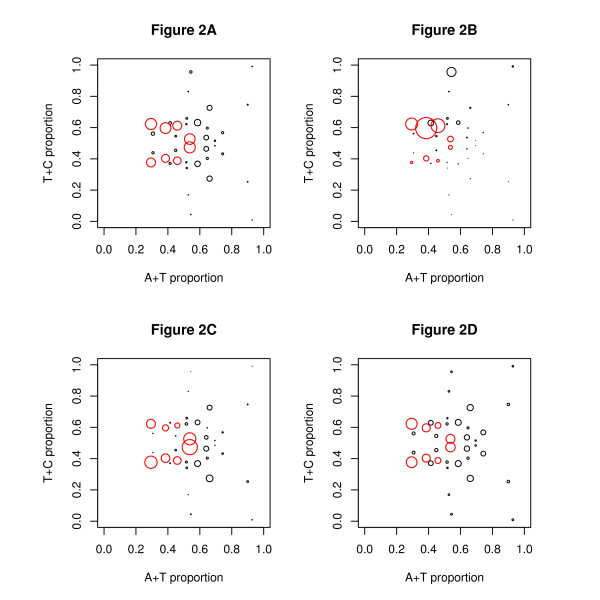
**Classes at binding sites and in the promoter regions**. The position of each mosaic class has been plotted as in Figure 1A. The area of each circle shows the proportion of binding sites in the class for (a) all 17 transcription factors (b) the transcription factor ZNF263 (c) the transcription factor SRF. Subplot (d) gives a comparison for bases in promoter regions (defined as the 1000 bases upstream of the TSS — the plot is based on all coding genes). The preferred classes are shown in red and contain (a) 75%, (b) 86% and (c) 79% of the binding sites. In (d) the preferred classes contain 60% of the bases--this percentage is high but not as high as for (a). The lack of symmetry in Figures 2B and 2C implies a preferred orientation/strand of the binding site within the class.

As explained in the Methods Section, we found the exact position of a set of experimentally verified transcription factor binding sites (TFBSs) and ran our model to discover which mosaic classes contained these sites. Detailed results will be presented for seventeen transcription factors and the average for these TFs is shown in Figure 2A. Four pairs of classes contain most of the binding sites: 2, 7, 9 and 14. We will refer to these classes as the "preferred classes" and they are shown in red in the Figures. Table 2 shows the proportion of sites in these classes for these transcription factors. The balance between the four pairs of classes varies with the factor and there may be a preferred orientation/choice of strand as may be seen from the example results for ZNF263 and SRF shown in Figures 2B and 2C. This tendency for binding sites to occur in these classes applies to every transcription factor — the lowest proportion of sites is 48% which is still nearly twice the proportion expected from the number of bases in these classes.

**Table 2:** Distribution of transcription factor binding sites across mosaic classes

Data Source	Factor	P	Sites	Pair 2	Pair 7	Pair 9	Pair 14	Total
Note 1	Note 2	Note 3	Note 4	Note 5	Note 5	Note 5	Note 5	Note 6
HAIB-K562	GABP	0.71	2557	0.054	0.035	0.113	0.774	0.976
HAIB-K562	NRSF	0.74	2006	0.236	0.231	0.254	0.142	0.862
HAIB-K562	SRF	0.64	367	0.370	0.083	0.111	0.229	0.794
YALE-GM128	NFKB	0.50	2653	0.322	0.156	0.139	0.069	0.686
YALE-HCT116	TCF7L2	0.50	3386	0.281	0.111	0.060	0.030	0.483
YALE-HepG2	SREBP1	0.50	4958	0.237	0.092	0.137	0.276	0.742
YALE-K562b	GATA1	0.52	3367	0.322	0.221	0.146	0.048	0.736
YALE-K562b	TR4	0.51	541	0.144	0.083	0.216	0.426	0.870
YALE-K562b	ZNF263	0.64	5098	0.049	0.194	0.466	0.147	0.856
YALE-K562	cFos	0.53	3746	0.287	0.186	0.111	0.018	0.603
YALE-K562	Max	0.60	3176	0.210	0.100	0.180	0.185	0.675
YALE-K562	NF-E2	0.81	4700	0.273	0.149	0.088	0.026	0.536
YALE-NT2D1	YY1	0.50	2967	0.252	0.135	0.157	0.333	0.876
YALE-K562-Ia30	STAT1	0.50	1039	0.398	0.104	0.077	0.059	0.638
ORegAnno	CTCF	1.00	4858	0.202	0.181	0.353	0.169	0.905
TRANSFAC	sp1	0.62	693	0.045	0.075	0.332	0.512	0.966
TRANSFAC	p53	0.91	608	0.266	0.203	0.081	0.021	0.571

Average of above				0.232	0.138	0.178	0.204	0.751

Genome proportions				0.134	0.071	0.042	0.009	0.256
Model proportions				0.134	0.069	0.042	0.009	0.255
Promoter region				0.170	0.069	0.128	0.233	0.600

1) For Encode data, this column gives the track, cell line and a possible note on the experimental protocol. For the HAIB data replication 1 was used. 2) Name of factor. 3) This column shows, *P*, the proportion of sequences for which MAST found a binding site. 4) The number sites found by MAST: later columns show the proportion of these sites in the class pair specified. 5) The values quoted are the average probabilities of the site being in the class(es); they are not maximum likelihood estimates. 6) Total of preceding columns. 7) Three lines of comparative figures are given. The line "genome proportions" gives the result of applying the analysis to 20 thousand bases chosen at random from the genome: the line "model proportions" gives those of the long term average of the HMM: the line "promoter region" gives the proportions found from applying the model to the bases within 1000 bases upstream of the transcription start site of all coding genes. The equality of "genome proportions" and "model proportions" is a cross check on the consistency of the calculations.

We ensured that in finding the positions of the binding sites we used the same motifs that had been previously reported. Logos for these motifs and cross references to previous work are given in Additional File 2. In the handful of cases where the known motif was not found the results were discarded.

We had little choice for which TF to use for a TF family and for which cell line to use for a TF, and where there is a choice, it is usually immaterial. This is shown in Table 3, which gives a table of ENCODE experiments for those TFs for which we have results. For consistency, the cell line K562 has been used where possible for the detailed analyses. (We discuss later whether the cell line influences the result.) Only for the ap1 family is there a real choice, where the c-Fos result for GM128 is an outlier from all the other results: here we have used the dataset (c-Fos/K562) that gave 60%.

**Table 3:** Summary of available experiments by cell line giving proportion of binding sites in the preferred classes

Factor	Cell line					
	**GM128**	**K562**	**HeLa**	**HepG2**	**NT2D1**	**HCT116**

GABP	0.991	0.976				
NRSF	0.844	0.862				
SRF	0.851	0.794				
JunD	0.293	0.687				
cFos		0.603	0.475			
cJun		0.602				
Max		0.675				
SREBP1				0.742		
SREBP2				0.734		
STAT1		0.638	0.519			
TR4		0.870	0.871	0.910		
GATA1		0.736				
GATA2		0.625				
NF-E2		0.536				
TCF7L2						0.483
Rad21		0.721				
NFKB	0.686					
YY1					0.876	
ZNF263		0.856				

This table summarises the ENCODE data for those transcription factors for which we found a motif from the MEME analysis and for which we had permission to use at the time of writing: it includes the JunD-GM128 result which is anomalously low. Other experimental treatments for cJun-K562 gave Ia6 = 0.637, Ig6 = 0.554, Ig30 = 0.573. For STAT1, the treatments were K562-Ia30 and HeLa-Ig30.

**Figure 3:**
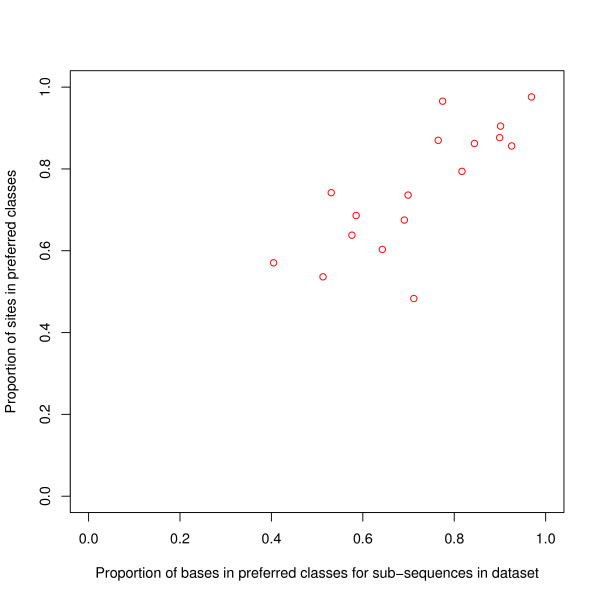
**Binding sites versus entire subsequences -- proportion of bases in the preferred classes**. For each transcription factor, the proportion of binding sites in the preferred classes has been plotted against the proportion of all bases in the preferred classes in all the sequences of the dataset. The latter proportion has been calculated for each sequence separately and then averaged over the sequences of the dataset. There is a strong relationship between the plotted variables: the correlation coefficient is 0.78.

We have also calculated the proportions of classes for bases for the whole length of the subsequences in the data. Given that this data is supposed to be enriched with TFBSs, (at least with one BS and more if BSs come in clusters), it would be a corollary of our results that these sequences would be enriched with the preferred classes. In fact, the proportion of bases in the preferred classes in these subsequences is very similar to that for the binding sites, (Figure 3). If it could have been assumed that binding sites were in typical positions on the subsequence, then this result implies that the main thrust of our results could have been deduced without knowing the exact position of the binding site within the subsequence.

The experimental procedure is to break the DNA near to and on each side of the TF binding site to give fragments that are a few hundred bases long. The subsequence finally reported as containing the binding site is defined by the position of the two ends estimated from the distribution of the genomic positions of the fragment ends. The neutral baseline sample of breakpoints, where a particular TF is not being "pulled down", is variously called the input signal or control library. The nature of the bias inherent in a control library has been discussed by [36], which observes biases from the copy number and from different kinds of repeat regions being under or over represented, but the most important bias they report is an enhancement near the transcription start site (TSS), especially for highly expressed genes. This later point is confirmed by [37,36] also make the point that the cell line may affect the bias in the control library. The reader might ask if any of these biases have affected our results.

**Figure 4:**
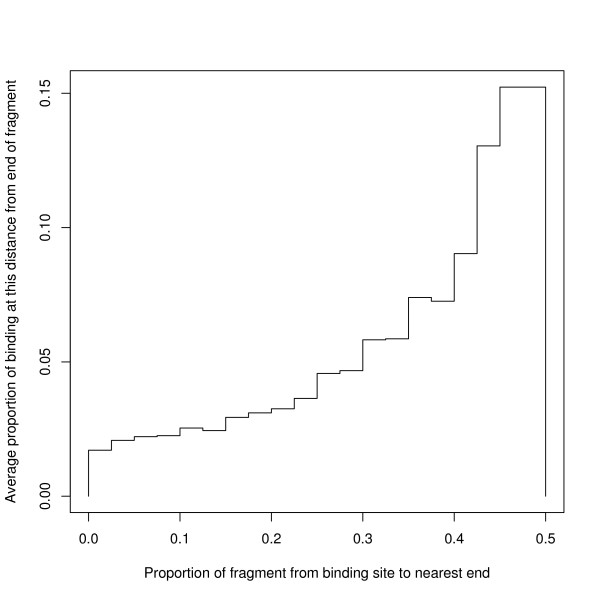
**Position of binding site within subsequence**. For each binding site, we have calculated *p *= the number of bases to the nearer end of the subsequence divided by the length of the subsequence; and for each dataset we have calculated the histogram of these proportions. The Figure shows the average heights of the histograms of individual datasets and shows a strong tendency for the binding site to be in the middle of the subsequence.

A number of general arguments indicate that our results are not affected by experimental bias. For 14 of the TFs analysed the experimental protocol included direct control for these biases [38,39] and the other experiments, that is for CTCF, sp1 and p53, also included their own checking procedures [2,40,41]. If there is any remaining bias in these experiments, it must be well behaved because the protocol produces uncontroversial motifs, as noted above. Although control libraries have a bias towards the TSS, as we discuss later, most of our binding sites are in fact far from the TSS, so that this aspect of the control bias is not represented in our final results. There is some evidence concerning TFBSs: DNAseI hypersensitive sites are usually taken to be indicative of regions containing a TFBS and reference [37] finds that there is a moderate enhancement for DNAseI hypersensitive sites near TSSs, but there is very little enhancement for distant sites. We also note that the binding site tends to be at the middle of the reported subsequence, Figure 4: that is away from any end effect which might be associated with the breakpoint itself. There is some complexity, because Auerbach *et al. *[37] suggest that protein binding in a region makes it more likely for that region to be enhanced in a control library and this would imply that TFBSs are more likely to occur in regions of higher tag density in the control library: and we have observed such a bias in the data — details not shown. However, we are confident that our results are true because of the following analysis. There are many TFBSs in regions where the tag density in the control library is low and we have analysed the mosaic classes of these TFBSs. In the data, the tag density is given in integral values and we have found the median value *M *of 10 k bases chosen at random [chromosome 1 was used to find *M*]: we then found the subset of TFBSs for which the tag density in the control library at the binding site was zero or strictly less than *M*. The results for the TFs in the cell lines K562/K56b from the YALE track show that the BSs from low tag density regions in the control data have the same character as the other BSs — see Table 4.

**Table 4:** Proportion of binding sites in the preferred classes for regions of low tag density in the control library

Factor	Number TFBS	Number TFBS	Ratio (3)/(2)	Proportion in preferred classes	Proportion in preferred classes
	**All**	**Low tag**		**All**	**Low tag**

**1**	**2**	**3**	**4**	**5**	**6**

GATA1	3367	1681	0.499	0.736	0.703
TR4	541	247	0.457	0.870	0.860
ZNF263	5098	3297	0.647	0.856	0.835
cFos	3746	1834	0.490	0.603	0.561
Max	3176	1382	0.435	0.675	0.614
NF-E2	4700	2322	0.494	0.536	0.489

Average of above			0.504	0.713	0.677

Column 2 gives the number of sites and column 5 the proportion of TFBSs in the preferred classes for all TFBS (as in columns 4 and 9 of Table 2). Columns 3 and 6 give this information for regions of low tag density in the control library, "sparse regions". Column 4 gives the proportion of sites in these regions. Note that the final column is very close to the preceding column and that often a substantial fraction of TFBSs are in "sparse regions". We conclude that the final results are not affected by experimental bias reflected in the control library. This analysis has been done for the "YALE" experiments using cell lines K562 and K562b. For K562, "sparse regions' were defined as a tag density of 0 or 1 in the input signal, and for K562b as a tag density of 0, 1 or 2.

To see if our results depend on the cell line, we have divided the ENCODE experiments into three groups: GM128, K562 and "Other". Many of the results are for K562, which appears to be a favourite cell line for this experiment. K562 is derived from cancerous cells, and it is plausible that this might affect the results. The other lines are also derived from cancer cells except for GM128 which has therefore been examined separately. Using a representative TF for each TF family, we calculated the mean proportion of sites in the preferred classes in each group of cell lines — see Table 5. Table 5 also shows the lower 95% confidence limit for a one tailed t-test. We conclude that each of these three groups of cell lines gives the same result and that our conclusions do not rely on any one cell line.

**Table 5:** Comparison between cell lines--total of preferred classes

Factor	Cell line		
	**GM128**	**K562**	**Other**

GABP	0.991	0.976	
NRSF	0.844	0.862	
SRF	0.851	0.794	
JunD	0.293		
cFos		0.603	0.475
Max		0.675	
SREBP1			0.742
STAT1		0.638	0.519
TR4		0.870	0.871
GATA1		0.736	
NF-E2		0.536	
TCF7L2			0.483
NFKB	0.686		
YY1			0.876
ZNF263		0.856	

Count	5	10	6
Average	0.733	0.755	0.661

Confidence Limit	0.477	0.674	0.503

This table gives a comparison between cell lines using a representative transcription factor for each family from Table 3. The last row gives the lower 95% confidence limit for the mean of the values in the column based on a one-sided t-test. The calculation includes the anomalously low value for JunD. We conclude that the choice of cell line does not affect the conclusion that TFBSs mainly occur in the preferred classes.

We now discuss if our results have a simple biological explanation. Most binding sites are far from the TSS: see [2] which reports only 22% of actual binding sites being within 1 kb of the TSS: compare the figure given by [1] of 53.5% of DNaseI hypersensitive sites as being further than 2500 bases from a TSS. For the TFs analysed in the current datasets, the proportion of actual binding sites further than 1500 bases from the TSS varies from TF to TF, but a typical figure is 80% to 90%, (Table 6, column 3). It is therefore not possible to explain our results by arguing that the proportions of classes found for binding sites reflect the proportions of classes in promoter regions. Sites far from the TSS (Table 6) show the same tendency as all sites to occur in the preferred classes (Table 2). For sites close to the TSS, an even higher proportion of sites, 87%, are in the preferred classes, but this result comes from the predominance of pair 14, which has a high proportion of CpG doublets (Table 7). There are, however, some points of similarity between the proportion of sites in the preferred classes and the proportion of bases in promoter regions — see Figure 2D — and we speculate that this could arise because promoter regions must be suitable for TFBSs.

**Table 6:** Distribution of transcription factor binding sites across mosaic classes for sites more than 1500 bases from a TSS

Data Source	Factor	Q	Sites	Pair 2	Pair 7	Pair 9	Pair 14	Total
Note 1	Note 2	Note 3	Note 4	Note 5	Note 5	Note 5	Note 5	Note 6
HAIB-K562	GABP	0.23	593	0.087	0.126	0.254	0.458	0.925
HAIB-K562	NRSF	0.85	1714	0.261	0.254	0.264	0.069	0.849
HAIB-K562	SRF	0.76	280	0.424	0.098	0.119	0.112	0.752
YALE-GM128	NFKB	0.85	2267	0.336	0.169	0.132	0.021	0.658
YALE-HCT116	TCF7L2	0.91	3075	0.280	0.116	0.057	0.012	0.466
YALE-HepG2	SREBP1	0.57	2844	0.283	0.132	0.135	0.065	0.615
YALE-K562b	GATA1	0.89	3011	0.330	0.231	0.139	0.021	0.721
YALE-K562b	TR4	0.35	191	0.144	0.162	0.322	0.110	0.738
YALE-K562b	ZNF263	0.80	4079	0.051	0.221	0.501	0.073	0.845
YALE-K562	cFos	0.94	3523	0.290	0.190	0.107	0.011	0.598
YALE-K562	Max	0.77	2453	0.237	0.121	0.193	0.065	0.615
YALE-K562	NF-E2	0.93	4380	0.275	0.152	0.086	0.011	0.524
YALE-NT2D1	YY1	0.61	1804	0.338	0.203	0.189	0.099	0.828
YALE-K562-Ia30	STAT1	0.86	898	0.414	0.111	0.067	0.019	0.611
ORegAnno	CTCF	0.90	4368	0.212	0.192	0.363	0.134	0.901
TRANSFAC	sp1	0.41	281	0.063	0.151	0.529	0.206	0.949
TRANSFAC	p53	0.95	579	0.271	0.210	0.075	0.004	0.560

Average of above				0.253	0.167	0.208	0.088	0.715

Genome proportions				0.134	0.071	0.042	0.009	0.256
Model proportions				0.134	0.069	0.042	0.009	0.255
Promoter region				0.170	0.069	0.128	0.233	0.600

Note 8) Column 3 gives the proportion, Q, of sites that are more than 1500 bases from a transcription start site, and column 4 gives the number of these sites. Other notes are as for Table 2.

**Table 7:** Distribution of transcription factor binding sites across mosaic classes for sites less than 1500 bases from a TSS

Data Source	Factor	Q	Sites	Pair 2	Pair 7	Pair 9	Pair 14	Total
Note 1	Note 2	Note 3	Note 4	Note 5	Note 5	Note 5	Note 5	Note 6
HAIB-K562	GABP	0.77	1964	0.045	0.007	0.071	0.869	0.991
HAIB-K562	NRSF	0.15	292	0.091	0.091	0.192	0.567	0.940
HAIB-K562	SRF	0.24	87	0.197	0.035	0.087	0.609	0.928
YALE-GM128	NFKB	0.15	386	0.238	0.081	0.184	0.347	0.850
YALE-HCT116	TCF7L2	0.09	311	0.295	0.062	0.089	0.207	0.652
YALE-HepG2	SREBP1	0.43	2114	0.174	0.036	0.141	0.560	0.911
YALE-K562b	GATA1	0.11	356	0.256	0.132	0.201	0.276	0.865
YALE-K562b	TR4	0.65	350	0.145	0.040	0.158	0.599	0.943
YALE-K562b	ZNF263	0.20	1019	0.041	0.089	0.329	0.443	0.902
YALE-K562	cFos	0.06	223	0.242	0.137	0.176	0.133	0.688
YALE-K562	Max	0.23	723	0.121	0.029	0.136	0.592	0.877
YALE-K562	NF-E2	0.07	320	0.249	0.106	0.120	0.225	0.699
YALE-NT2D1	YY1	0.39	1163	0.119	0.029	0.107	0.696	0.951
YALE-K562-Ia30	STAT1	0.14	141	0.298	0.061	0.140	0.313	0.812
ORegAnno	CTCF	0.10	490	0.110	0.081	0.258	0.486	0.935
TRANSFAC	sp1	0.59	412	0.033	0.024	0.198	0.722	0.977
TRANSFAC	p53	0.05	29	0.166	0.062	0.194	0.365	0.787

Average of above				0.166	0.065	0.164	0.471	0.865

Genome proportions				0.134	0.071	0.042	0.009	0.256
Model proportions				0.134	0.069	0.042	0.009	0.255
Promoter region				0.170	0.069	0.128	0.233	0.600

Note 8) Column 3 gives the proportion, Q, of sites that are less than 1500 bases from a transcription start site, and column 4 gives the number of these sites. Other notes are as for Table 2.

**Figure 5:**
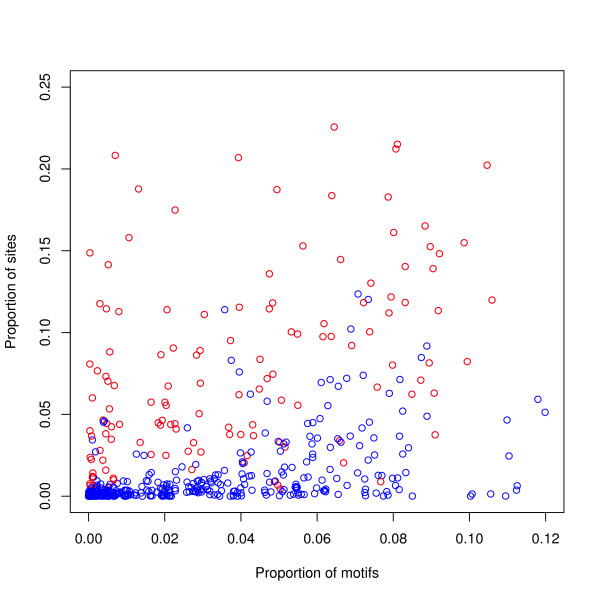
**Proportion of binding sites versus proportion of motifs for individual classes**. For each of the 17 transcription factors, the proportion of binding sites in each mosaic class has been plotted against the proportion of motifs found in this class. The preferred classes have been plotted in red and the other classes in blue. As there are 19 pairs of classes, (4 pairs of preferred classes and 15 pairs of non-preferred classes), each transcription factor contributes 8 red points and 30 blue points to the graph. There is a correlation between the variables (Spearman coefficient = 0.68), but this comes from the mass of points near the x-axis, so that the proportion of motifs is not a useful predictor of the proportion of sites. Not plotted is a red point outlier at (0.01, 0.62).

**Figure 6:**
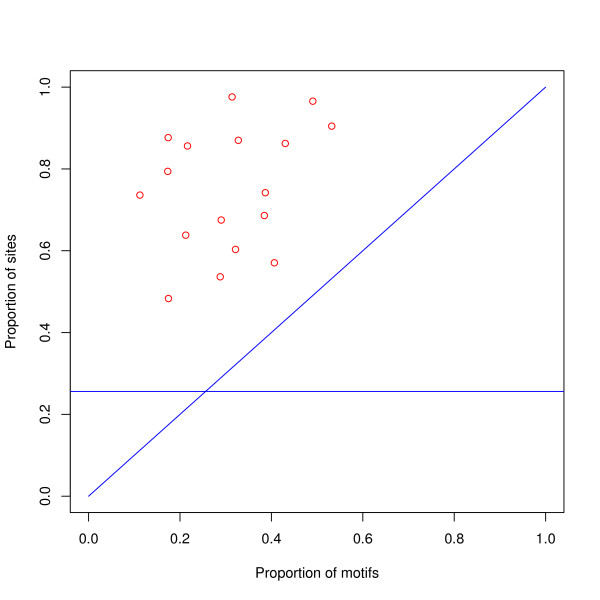
**Proportion of binding sites versus proportion of motifs in the preferred classes**. For each of the 17 transcription factors, the proportion of binding sites in the preferred classes has been plotted against the proportion of motifs in these classes. The height of the horizontal line gives the proportion of all bases in the genome in these classes. The Figure shows that for the preferred classes the proportion of sites is greater than the proportion of motifs: it also shows that the proportion of motifs is not a good predictor of the proportion of sites for these classes. It can also be seen that the proportion of sites is greater than the proportion of the genome in the preferred classes.

Another trivial explanation of our results might be that subsequences are generated at random within each class and the preferred classes shown in Table 2 are merely those classes which are most likely to generate the motif of the factor. This explanation is not likely to work for as many as seventeen factors, but this possibility has been tested as follows. For each transcription factor we used the motif found by MEME in finding the exact binding site positions, and counted the number of occurrences of this motif in artificial sequences representative of each class — see the Methods Section for details. The proportion of occurrences of motifs in each class is not a useful predictor of the proportion of sites in each class, neither for individual classes (see Figure 5), nor for the total in the preferred classes (see Figure 6). More detailed results are given in Table 8.

**Table 8:** Distribution of motifs across mosaic classes

Transcription factor	Mosaic classes				
	**Pair 2**	**Pair 7**	**Pair 9**	**Pair 14**	**Total**

GABP	0.133	0.102	0.058	0.021	0.314
NRSF	0.176	0.148	0.096	0.011	0.430
SRF	0.131	0.033	0.009	0.001	0.173
NFKB	0.211	0.108	0.058	0.008	0.385
TCF7L2	0.125	0.039	0.009	0.001	0.175
SREBP1	0.198	0.101	0.074	0.014	0.387
GATA1	0.079	0.027	0.006	0.001	0.112
TR4	0.180	0.097	0.046	0.005	0.328
ZNF263	0.055	0.099	0.052	0.010	0.216
cFos	0.183	0.094	0.042	0.002	0.322
Max	0.141	0.079	0.060	0.010	0.290
NF-E2	0.159	0.090	0.037	0.002	0.288
YY1	0.111	0.045	0.016	0.002	0.174
STAT1	0.160	0.039	0.012	0.002	0.213
CTCF	0.159	0.168	0.164	0.041	0.532
sp1	0.082	0.152	0.182	0.074	0.491
p53	0.182	0.126	0.086	0.013	0.406

Average of above	0.145	0.091	0.059	0.013	0.308

This table gives the proportion of motifs in the class pair for each transcription factor: the value has been derived from artificial sequences and adjusted for the relative proportion of bases in each class in the human genome. This table is to be compared with Table 2 which gives the distribution of actual binding sites.

Using the data plotted in Figure 6, we find the following statements to be statistically significant. The proportion of sites in the preferred classes is greater than the proportion of bases in the genome: a one-sided T-test gives n = 17, df = 16, t = 13.4, p = 2.0e-10, and the 95% lower confidence limit of average proportion of sites = 0.68 (~0.43 more than the proportion of bases). The difference between the proportion of sites and the proportion of motifs in these classes is greater than zero: a one-sided T-test gives n = 17, df = 16, t = 11.2, p = 2.7e-9, and the 95% lower confidence limit of average difference = 0.37. On the other hand, a comparison between the proportion of motifs and the proportion of bases has a much higher p-value:- a one-sided T-test gives n = 17, df = 16, t = 1.8, p = 0.047, and the 95% lower confidence limit of average proportion of motifs = 0.257 (to be compared with the proportion of bases, 0.256). A prior Shapiro-Wilk test did not show any departure from normality for the samples: the sample of site proportions gave p = 0.54 and for the motif proportions p = 0.75.

## Conclusions

We find that the preferred mosaic classes contain 75% of experimentally verified binding sites for transcription factors from seventeen families of transcription factors. The same mosaic classes constitute 26% of the genome and contain only 31% of the motifs for these factors. These results are shown in Figure 6. This method of analysis will help with the problem of false positive binding sites found by computational methods. These results must mirror the biological and physical processes involved and imply a profound difference between the mosaic classes.

Recent research shows that biologically effective binding sites have a range of binding affinities which lead to a corresponding range of gene expression levels [42,43]. It would be interesting to see if the mosaic class which contains a transcription factor binding site also affects the expression level.

Another area for research would be to repeat the analysis for a defined subset of the genome to see if a different set of mosaic classes emerged. One interesting result would be if some parts of the genome showed mosaic classes preserved from an earlier stage of evolution — for example before CpG doublets degraded to CpA doublets. It would also be instructive to perform similar analyses using a large scale biological feature to identify a preferred strand. We have done some exploratory work on the approximate symmetries shown in Figure 1, and we think there is more to be said on this subject.

## Methods

### The mathematical framework

Our results on mosaic classes come from using a Hidden Markov Model (HMM) to model a large sample of the human genome. The analysis is entirely independent of any data on the position of transcription factor binding sites. In the HMM, each mosaic class has been modeled as a single state which implies that the length distribution in the model will be a simple exponential distribution. The probability of a base has been taken to depend on the class and the immediately previous base. The model was trained using the Expectation-Maximisation (E-M) algorithm — references and formulae are given in Additional File 3. The training was continued until convergence was achieved for the proportion of the genome represented by each class and for the base proportions within each class. All emission and transition probabilities may have different values when the model is trained.

Classes have been matched in pairs, with the emission probabilities of the paired classes showing A/T and C/G symmetry. This symmetry also means that the transition probability between two classes is the same as that between their matched counterparts. These symmetries have been imposed at initialisation and maintained at each iteration.

Variability in the analyses comes from two sources: the initialisation of the HMM and the choice of sequence used to train the HMM. The variability has been controlled by using eight replications with different initialisation and different training sequences, with each replication using 6000 sequences of 2000 bases. These sequences were taken at random positions from the whole genome, assembly NCBI36, taken from Ensembl [44].

A common subset of classes from these replications was obtained as follows. The classes were considered in the space defined by the three coordinates T+A, T+C, T+G as defined by the steady-state model average and classes of different replication runs were matched if they were close together in this space. Classes were put into sets, so that each set contained one class from each run, with the aim of firstly maximising the number of sets and secondly minimising the average distance between classes within a set. A class was not put into a set unless it could be put into a set where it was close to other classes in the set. The procedure was as follows: in step one, the classes from runs 1 and 2 were matched and the position of each resulting pair was averaged: in the second step these positions were then matched with the classes from run 3: this step was repeated with the average position from the previous step being matched with the classes of the next run. Matching was only allowed between classes (or positions) within a distance, (*D*, taken as 0.12) in the base-proportion-space, but within that constraint, at each step, the algorithm made a complete search of how the classes should be matched to find the optimum in terms of number of sets and average distance. When finding the average position of pairs of classes, weights were used so that each run made an equal contribution to the final average. This procedure does not guarantee the global optimum and the heuristic was used of taking the best result when the runs were arranged in different orders: we used (8 × 7 × 6 × 5) orders: that is all the orders of the replication runs which differ in the first four places.

When a common subset of classes had been obtained the emission probabilities and transition probabilities of the matched classes were averaged and strand symmetry of paired classes reimposed. This average-model was used to initialise a final round of training using the same 48 thousand sequences of 2000 bases as were used in the initial eight replications. The parameters of the final HMM are given in Additional File 1.

This protocol was designed after earlier experiments in which classes were merged during the HMM training if they came close together in the space of base proportions. These experiments suggested that there were around three dozen classes and therefore to allow for some spurious classes in some runs, 50 classes were used to initialise the eight replications.

**Figure 7:**
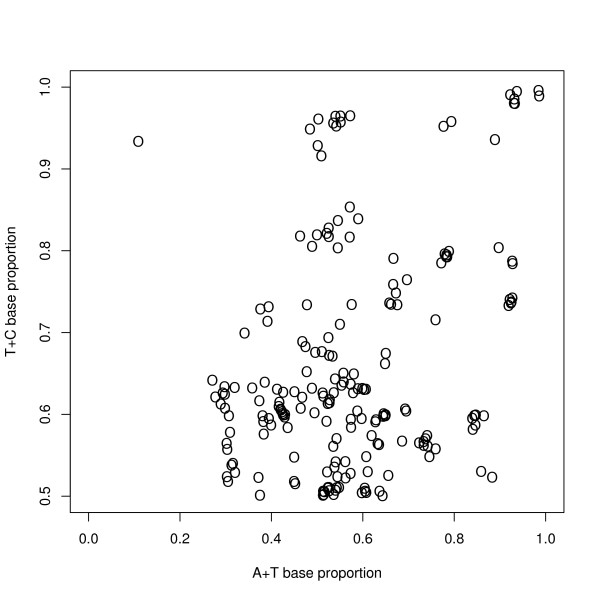
**Classes from constituent runs — T+C versus A+T**. The classes found from each of the 8 preliminary replication runs have been overlaid on the same plot. The classes have been plotted by their A+T and T+C content as in Figure 1A, except that the figure includes only classes with T+C > 0.5 — the omitted classes mirror those shown. The plot shows a strong similarity between the results of the replication runs, showing the reproducibility of the method--a point taken up in Figure 8. Each replication run was randomly initialised with 25 pairs of matched classes, and trained on 6000 sequences of 2000 bases taken at random from the human genome.

**Figure 8:**
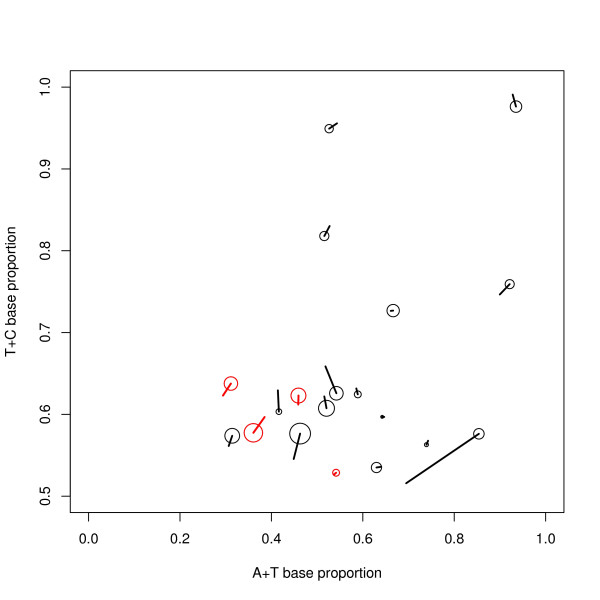
**Uncertainty in the A+T and T+C proportions of the mosaic classes**. The circles show the position of the classes used to initialise the final HMM training run. The radius of these circles is the standard error of this initial position calculated from the positions of the classes averaged to produce these initial classes--compare Figure 7. The line from each circle shows the position of the class after the final HMM training run, which is also shown in Figure 1A. The figure shows only the region T+C > 0.5--the omitted region mirrors the region shown. The statistical robustness of the method for deriving the mosaic classes is demonstrated by i) the consistency of the results of the preliminary runs shown by the small size of the circles and ii) the small difference between the classes at the beginning and end of the final training run shown by the short length of the lines.

To give an indication of the statistical robustness of the method, Figure 7 shows the results of the eight constituent runs. This Figure shows that the classes are well defined. It also shows — as is to be expected — that using different sequences for the analysis will affect the results. Figure 8 gives a quantitative demonstration of statistical robustness. This Figure shows the position of the classes in the average-model, and an estimate of the uncertainty in these positions. This uncertainty is shown by the radius of the circles and can be seen to be small: the uncertainty has been calculated analogously to the standard error to the mean, as the root mean square distance from the composite-class to the constituent classes divided by the square root of the number of classes. Figure 8 also shows the position of the final classes after the final training run: in general the final position is near the initial position.

### Finding classes for the Binding Sites

The positions of subsequences containing binding sites was obtained from the UCSC browser [45] using tracks created for the ENCODE project [1] and the tables labeled "peaks":- for three factors from the "HAIB TFBS" data [38], (ENCODE November 2008 and February 2009 freezes) and for eleven factors from the "YALE TFBS" data [39], (release 2 of October 2009). We also used data [40] for CTCF from the ORegAnno database (version 17th September 2008) [46], and for sp1 [2] and p53 [2,41]: the data for sp1 and p53 was extracted from TRANSFAC [47] professional version 11.4 and the flanking bases added by TRANSFAC were removed. (For the ENCODE and CTCF data, if there were more than 5000 sequences in a dataset then about 5000 sequences were chosen evenly from the dataset for further analysis. Sequences longer than 700 bases were then excluded: sequences less than 30 bases were extended by 15 bases on each side. Sequences were also excluded from the masked data if more than a quarter of the bases were masked. In some cases the motif was found from a different subset of sequences than later used to find the binding sites.) The genomic coordinates of these subsequences were used to obtain the RepeatMasked sequence [48] from Ensembl [44], which was then analysed by MEME [10,49] to find the binding site motif of the transcription factor. MEME generated three motifs for consideration. Logos for these motifs were drawn with Weblogo [50] and checked against logos previously reported. (MEME does not find the motif for sp1 unaided and was initialised with the motif CCCCGCCCCC.) Using the version of the known motif found by MEME and the corresponding unmasked sequences, the positions of the binding sites within these sequences were found by MAST, a companion tool of MEME. MAST needs a parameter, *mt*, the threshold for the p-value, to determine how many sites will be reported and we normally used the default, (0.0001), for this. However, in some cases this procedure only finds a motif in a small proportion of sequences, and if necessary the value of *mt *was increased so that a motif was found in at least half the sequences. In fact, the final results are largely unaffected by the choice of *mt*. The position of all sites found by MAST in the subsequences was then cross referenced to the position in the genome.

The non-masked sequence of length 4000 bases with the motif in the middle was analysed using the final HMM model to determine the class in which the motif occurred. For this we calculated the probability (of a base being in each class) as in the expectation step of the EM-algorithm. This is not the same as using the maximum likelihood estimate (as in the Viterbi algorithm) of the class containing the binding site to calculate the class proportions. For the classes selected for Table 2, the maximum likelihood estimates are slightly higher than those shown, and lead to a slight (apparent) discrepancy between the theoretical model distribution and the observed genome distribution.

Analyses of promoter regions are based on all coding genes as defined by Ensembl 52.

### Estimating proportion of motifs by class

For each class, the HMM was used to generate artificial sequences (10 sequences of 1 million bases). For each transcription factor, MAST [10], a sister tool of MEME, was used to identify and count positions of the motif in the artificial sequences using its default parameters and the motif found by MEME. The proportion of motifs in each class was calculated in proportion to *a *× *b *× *c *where *a *= the number of bases in the genome, *b *= the proportion of all bases in the mosaic class, and *c *= the number of motifs per 10 million bases found from the MAST analysis.

## Supplementary Material

Additional file 1 **Additional file**1**is a text file giving the parameters of the final HMM. Fields are defined by comments in the file. Classes *a *and *b *of class pair *n *correspond to states 2*n *- 1 and 2*n *in the file.** Click here for file

Additional file 2 **Additional file**2**is a pdf file giving logos for the motifs found by MEME in our analysis and cross references to previous reports of these motifs.** Click here for file

Additional file 3 **Additional file**3**is a pdf file giving references and formulae for the EM-algorithm.** Click here for file
