# Supplementary material for: Most transcription factor binding sites are in a few mosaic classes of the human genome
Source: BMC Genomics. 2010 May 6;11:286. doi: 10.1186/1471-2164-11-286 (PMC2881025; doi:10.1186/1471-2164-11-286)
Supplement: Additional file 2 — Additional file2is a pdf file giving logos for the motifs found by MEME in our analysis and cross references to previous reports of these motifs. [file 1471-2164-11-286-S2.PDF]

Logos of motifs used in the main analyis

Kenneth J Evans*

School of Crystallography, Birkbeck College, University of London, Malet Street, London, WC1E 7HX, UK

Email: Kenneth J Evans*- k.evans@mail.cryst.bbk.ac.uk;

*Corresponding author

Introduction

As described in the methods section of the main text, MEME was used to find the motifs of transcription factor binding sites and these motifs were then used to find the exact position of the binding sites in the sequences. Figures 1 to 17 give the logos of the motifs corresponding to the results shown in Table 2 of the main text. The notes to these figures give cross references to previous reports of these motifs. Logos have been drawn with Weblogo [1].

In those cases where the paper quotes results for these transcription factors for other cell lines—for example in Tables 3 and 5—the motifs have not been reported but are are very similar to those given here.

Figure 1: Logo of motif used for GABP — This motif corresponds to motif MA0062.2 in the core JASPAR collection [2].

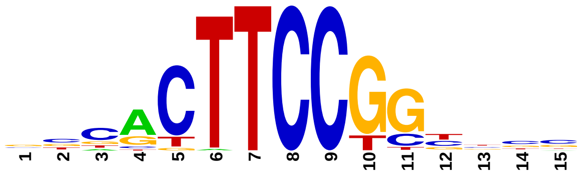

Figure 2: Logo of motif used for NRSF — This motif corresponds to motif MA0138.2 in the core JASPAR collection [2].

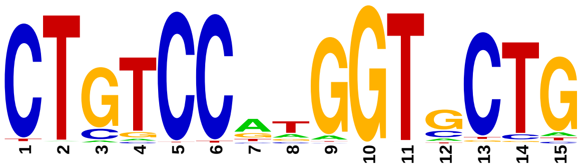

Figure 3: Logo of motif used for SRF — This motif corresponds to motif MA0083.1 in the core JASPAR collection [2].

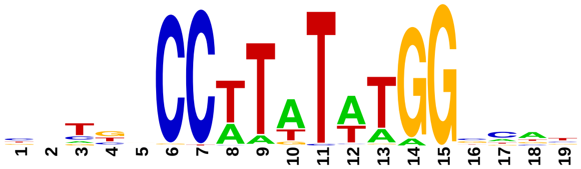

Figure 4: Logo of motif used for TCF7L2 — Compare the sites given for factor T02918 in TRANSFAC public version 7.0 [3]. This factor is also called TCF4.

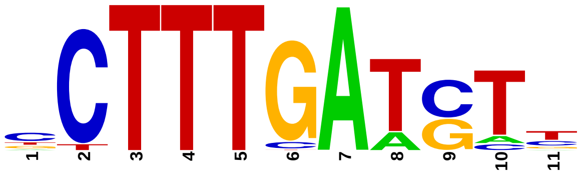

Figure 5: Logo of motif used for STAT1 — This motif corresponds to motif MA0137.2 in the core JASPAR collection [2].

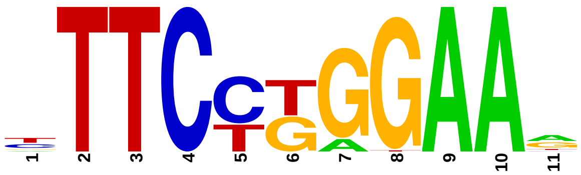

Figure 6: Logo of motif used for SREBP1 — This motif is not the SRE or E-box motif normally quoted (see for example SwissProt), but is the one given by [4].

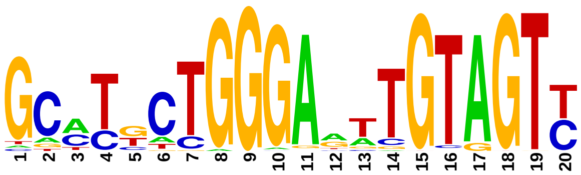

Figure 7: Logo of motif used for NF-E2 — This motif corresponds to motif MA0150.1 in the core JASPAR collection [2].

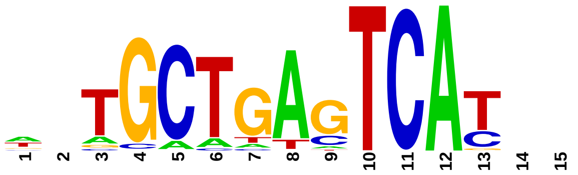

Figure 8: Logo of motif used for ZNF263 — This motif looks a degenerate low complexity motif but is close to the one given by [5].

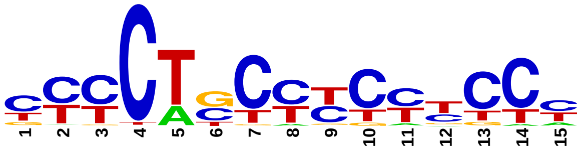

Figure 9: Logo of motif used for GATA1 — This motif corresponds to motifs MA0140.1 and MA0035.2 in the core JASPAR collection [2].

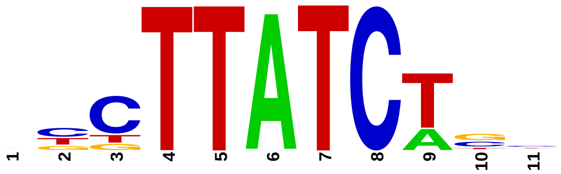

Figure 10: Logo of motif used for Max — This motif corresponds to motif MA0058.1 in the core JASPAR collection [2].

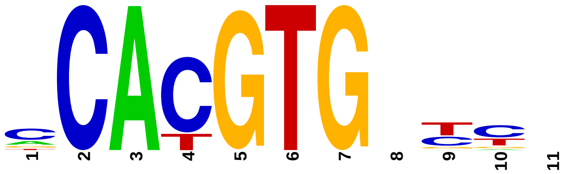

Figure 11: Logo of motif used for c-Fos — This motif corresponds to motif MA0099.2 for ap1 in the core JASPAR collection [2].

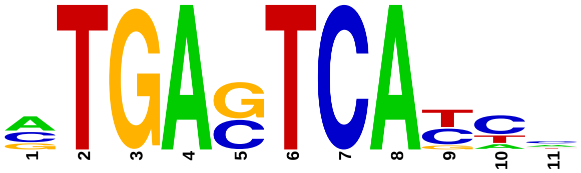

Figure 12: Logo of motif used for p53 — This is very similar to the logo given in [6]: it is also similar to motif MA0106.1 in the core JASPAR collection [2].

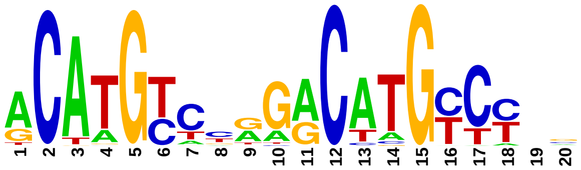

Figure 13: Logo of motif used for sp1 — This motif corresponds to motif MA0079.2 in the core JASPAR collection [2].

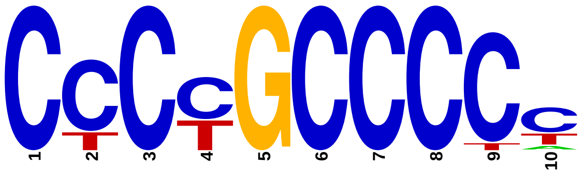

Figure 14: Logo of motif used for CTCF — This motif corresponds to motif MA0139.1 in the core JASPAR collection [2].

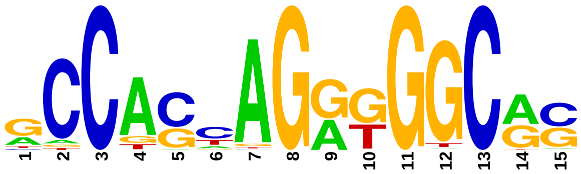

Figure 15: Logo of motif used for TR4 — One half of this motif is given in [7].

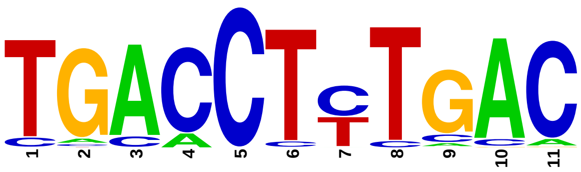

Figure 16: Logo of motif used for YY1 — This motif corresponds to motif MA0095.1 in the core JASPAR collection [2].

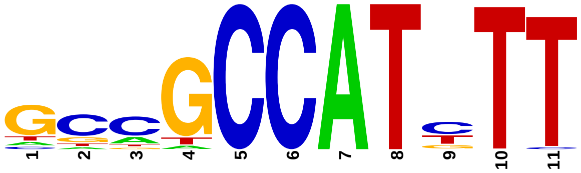

Figure 17: Logo of motif used for NFKB — This motif corresponds to motif MA0105.1 in the core JASPAR collection [2].

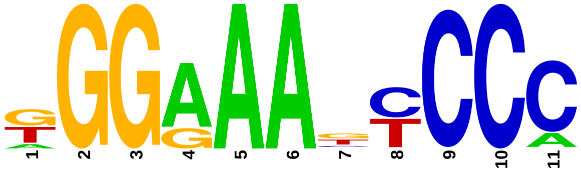

References

1. Crooks GE, Hon G, Chandonia JM, Brenner SE: **WebLogo: A sequence logo generator**. *Genome Research* 2004, **14**:1188–1190.

2. Bryne JC, Valen E, Tang MH, Marstrand T, Winther O, da Piedade I, Krogh A, Lenhard B, Sandelin A: **JASPAR, the open access database of transcription factor-binding profiles: new content and tools in the 2008 update**. *Nucleic Acids Res* 2008, **36**:D102–106.

3. Matys V, Kel-Margoulis O, Fricke E, Liebich I, Land S, Barre-Dirrie A, Reuter I, Chekmenev D, Krull M, Hornischer K, Voss N, Stegmaier P, Lewicki-Potapov B, Saxel H, Kel A, Wingender E: **TRANSFAC® and its module TRANSCompel®: transcriptional gene regulation in eukaryotes**. *Nucleic Acids Res* 2006, **34**:D108–D110, [www.gene-regulation.com/pub/databases.html].

4. Seo YK, Chong HK, Infante AM, Im SS, Xie X, Osborne TF: **Genome-wide analysis of SREBP-1 binding in mouse liver chromatin reveals a preference for promoter proximal binding to a new motif**. *Proc Natl Acad Sci USA* 2009, **106**:13765–13769.

5. Frietze S, Lan X, Jin VX, Farnham PJ: **Genomic targets of the KRAB and SCAN domain containing zinc finger protein 263 (ZNF263)**. *J Biol Chem* 2009. [M109.063032].

6. Wei CL, Wu Q, Vega VB, Chiu KP, Ng P, Zhang T, Shahab A, Yong HC, Fu Y, Weng Z, Liu J, Zhao XD, Chew JL, Lee YL, Kuznetsov VA, Sung WK, Miller LD, Lim B, Liu ET, Yu Q, Ng HH, Ruan Y: **A global map of p53 transcription-factor binding sites in the human genome.** *Cell* 2006, **124**:207–219, [http://dx.doi.org/10.1016/j.cell.2005.10.043].

7. Hwang SB, Burbach JPH, Chang C: **TR4 orphan receptor crosstalks to chicken ovalbumin upstream protein-transcription factor and thyroid hormone receptor to induce the transcriptional activity of the human immunodeficiency virus type 1 long-terminal repeat**. *Endocrine* 1998, **8**:169–175.
