# Supplementary material for: Most transcription factor binding sites are in a few mosaic classes of the human genome
Source: BMC Genomics. 2010 May 6;11:286. doi: 10.1186/1471-2164-11-286 (PMC2881025; doi:10.1186/1471-2164-11-286)
Supplement: Additional file 3 — Additional file3is a pdf file giving references and formulae for the EM-algorithm. [file 1471-2164-11-286-S3.PDF]

The formulae for the E-M algorithm applied to Hidden Markov Models

Kenneth J Evans*

School of Crystallography, Birkbeck College, University of London, Malet Street, London, WC1E 7HX, UK

Email: Kenneth J Evans*- k.evans@mail.cryst.bbk.ac.uk;

*Corresponding author

Background

The Expectation-Maximisation algorithm is an iterative algorithm for estimating parameters in a statistical model where the observations are incomplete. Each iteration consists of two steps: an E-step or Expectation-step followed by an M-step or Maximisation-step. The classic paper is by Dempster, Laird and Rubin [1] which generalised a number of special cases. The application to Hidden Markov Models is itself a special case and here the algorithm is called the forward-backward algorithm, as the E-step requires a forward pass and then a backwards pass through the observations. It is also known as the Baum-Welch algorithm after the authors who first worked on the method [2]. Tutorials on the method include [3–5]. The formulae given here follow some variations given by [6]. In practice I have largely followed the equations of [7]—work which was later published as [8]. Readers interested in the underlying theory are referred to the mathematics in these references: this note gives only the operational formulae which are comparatively straightforward.

Structure of the problem

A Markov Model is one which models a random walk from one state to another. The model is stochastic, and the probability of the system being in state j at time t depends only on the state the system was in at time $(t-1)$—not for example on the time or states before time $(t-1)$. The Markov Model is therefore

fully defined by the transition probabilities and a statement about the initial conditions.

A Hidden Markov Model (HMM) is a Markov Model where the states cannot be observed directly but at each time step the system gives a signal which can be observed. These signals are usually called "emissions". These emissions depend only on the state of the system at that time. Therefore a Hidden Markov Model is defined by the parameters of the Markov Model and the emission probabilities. In practice, different states can emit the same signals so that to deduce the states from the emissions is a problem in statistical inference.

We have described the Markov Model as a random walk in time: in most applications this is the natural interpretation, but in the present case the position in the sequence is the equivalent of time and the bases in the sequence are the emissions.

Standard formulae

Suppose our data sequence is n bases long: let t label the position in the sequence so that t runs from 1 to n. Let the base at position t be y_t: in our application y_t is the known observed data. In a Hidden Markov Model the number of states, N, is assumed to be known. Let i label the states so that i can take the values 1 to N.

The unknown parameters of the model are:- the transition probabilities $T(i, j)$, where $T(i, j)$ is the probability of the state being j at a given position if the state is i at the previous position; and the emission probabilities $E(i, b)$, where $E(i, b)$ is the probability of emitting base b in state i; and the (prior) probabilities $A(i)$ that the system is in state i at the first position.

Given the sequence y_t and the value of N, the E-M algorithm gives a method of estimating (a) the probabilities of the system being in each state at each position and (b) the unknown parameters of the model. The algorithm procedes iteratively beginning with trial values for the unknown parameters. In the E-step the state probabilities are updated. This is the probability $\gamma(i, t)$ of the system being in state i at position t. In the M-step the estimates for the unknown parameters are updated. The E-step is so-called because it calculates the expected values of these probabilities: the M-step is so-called because it finds a

maximum likelihhod estimate of the parameters.

In the formulae that follow, i and j label states, t labels the position in the sequence, and b labels the base.

The Expectation-step consists of the forward pass and the backward pass. The equations for the forward pass are as follows: the quantities are calculated in the order of increasing t, beginnning with $t = 1$.

$$\text{For all } j : \alpha'(t = 1, j) = A(j)E(j, y_{t=1}) \tag{1}$$

$$\text{For all } j, t > 1 : \alpha'(t, j) = \sum_i \alpha(i, t-1)T(i, j)E(j, y_t) \tag{2}$$

$$\text{For all } j, t : \alpha(t, j) = \alpha'(t, j)/\sum_j \alpha'(t, j) \tag{3}$$

$$\text{For all } i, j, t : \xi'(t, i, j) = \alpha(t-1, i)T(i, j)E(j, y_t) \tag{4}$$

$$\text{For all } i, j : \xi(t, i, j) = \xi'(t, i, j)/\sum_{ij} \xi'(t, i, j) \tag{5}$$

The normalisations in equations 3 and 5 are required to prevent underflow when the calculations are performed on a computer.

The equations for the backward pass are as follows: the quantities are calculated in the order of decreasing t, beginnning with $t = n$.

$$\text{For all } j : \gamma(t = n, j) = \alpha(t = n, j) \tag{6}$$

$$\text{For all } i, j, t > 1 : \zeta(t, i, j) = \xi(t, i, j)\gamma(t, j)/\alpha(t, j) \tag{7}$$

$$\text{For all } i, t > 1 : \gamma(t-1, i) = \sum_j \zeta(t, i, j) \tag{8}$$

In the Maximisation-step, the parameters of the model are updated:

$$\text{For all } i : A(i) = \gamma(t = 1, i) \tag{9}$$

$$\text{For all } i, j : \eta(i, j) = \sum_{t>1} \zeta(t, i, j) \tag{10}$$

$$\text{For all } i, j : T(i, j) = \eta(i, j)/\sum_j \eta(i, j) \tag{11}$$

$$\text{For all } i, b : E'(i, b) = \sum_{t, b=y_t} \gamma(t, i) \tag{12}$$

$$\text{For all } i, b : E(i, b) = E'(i, b)/\sum_b E'(i, b) \tag{13}$$

Application to the paper

A number of minor changes were made when using these formulae in the current application. The probability of a given base at a given position was assumed to depend both on the state in the Markov Model and the previous base. This gives a number of minor book keeping changes to the formulae: The length of the sequence of doublets is one less than the number of bases, and $t = 1$ refers to the second base of the sequence and $t = n$ to the last. E is now a function of three variables: i the state, a the previous base, b the emitted base. Equation 12 becomes

$$\text{For all } i, b : E'(i, a, b) = \sum_{t, a=y_{t-1}, b=y_t} \gamma(t, i) \tag{14}$$

and equation 13:-

$$\text{For all } i, a, b : E(i, a, b) = E'(i, a, b) / \sum_b E'(i, a, b) \tag{15}$$

As explained in the main text, states have been matched in pairs with states in each pair showing strand symmetry. This has consequences for the transition probabilities and the emission probabilities. Let state i be matched to i' and j to j'. For the transition probabilities the values from equation 11 were updated by replacing both $T(i, j)$ and $T(i', j')$ by $(T(i, j) + T(i', j'))/2$.

Keeping the symmetry between the emission probabilities is more subtle: if base b' is the complement base to base b and a' to a, then it is *not* in general true that the emission probability of base a following base b in state i is the same as the probabilitiy of base b' following base a' in state i'. The method used was based on the fact that under strand symmetry a doublet (ab) in class i should occur as often as doublet $(b'a')$ in class i' does on the same strand. The E from equation 15 was used to calculate the relative frequency of each doublet in each class. This was done by calculating proportion, $P(a)$, each base, a, in each class i iteratively:- Let z label the iteration, put $P(a, z = 1) = 1/4$, put $P(b, z) = \sum_a P(a, z-1)E(i, a, b)$ and iterate for 5000 times. The probability of doublet (ab) in class i is then $P(a)E(i, a, b)$. Let x be the proportion of doublets (ab) in class i calculated by this method and x' the proportion of doublets $(b'a')$ in class i'. We then argue that the best estimate of what these proportions ought to be for each of these classes is $f(ab) = (x + x')/2$. Hence, the probability, $E(i, a, b)$ that base b follows base a in class i which was calculated from equation 15 is updated to the value, $f(ab)/\sum_b f(ab)$.

The prior probability of the first base being in state i was estimated from the transition probabilities

$T(i,j)$, using an iterative procedure:- Put $Q(i,z=1)=1/N$ and put $Q(j,z)=\sum_i Q(i,z-1)T(i,j)$ and iterate 5000 times. $A(i)$ is put equal to the final value of $Q(i,z)$.

Analysing a sequence

Once the parameters of the HMM have been estimated it is possible to calculate the probabilities of the base at position t being emitted from state i. This is calculated with a single use of the E-step of the EM-algorithm.

However, the more intuitive question is the different question of "What is the most likely path in the Markov Model to explain this sequence?" where "path" is the set of states I_t at each position t in the sequence which maximises the likelihood. This question is answered by the Viterbi algorithm—the original reference is [9]. The paper does not quote results using this algorithm but we give the formulae for completeness.

Consider the best path up to position t which ends in state i: "best path" means the one with highest likelihood: let $\delta(t,i)$ be the likelihood of this path. For the first position:

$$\text{For all } j : \delta(t=1,j) = A(j)E(j,y_{t=1}) \tag{16}$$

There are N paths up to time t, one for each final state i. We now ask which is the best path up to state j at time $t+1$. This must be one of the N paths just mentioned followed by the path i to j, and likelihood of this best path to state j at time $t+1$ will be given by

$$\text{For all } j : \delta(t+1,j) = \max_i\{\delta(t,i)T(i,j)E(j,y_{t+1})\} \tag{17}$$

Hence we have a recurrence relation for δ. In making this calculation we also make a note of which i was used to get to j at time $t+1$ which we define as $\phi(t+1,j)$. Again to avoid underflow in the computer calculation, it is convenenient to normalise the $\delta(t+1,j)$ before proceding to the next time step.

Hence the state I_n of the last base in the most likely path is the state j which maximises $\delta(n,j)$. The corresponding value of δ is the likelihood of the maximum likelihood path. The values of ϕ can then be

used to retrace the path, that is to find the state at each previous position:

$$I_t = \phi(t+1, I_{t+1}) \tag{18}$$

References

1. Dempster A, Laird NM, Rubin DB: **Maximum Likelihood from Incomplete Data via the EM Algorithm**. *Journal of the Royal Statistical Society Series B (Methodological)* 1977, **39**:1–38.
2. Baum LE, Petrie T, Soules G, Weiss N: **A maximization technique occurring in the statictical analysis of probabilistic functions of Markov chains**. *Ann Math Stat* 1970, **41**:164–171.
3. Rabiner LR: **A tutorial on hidden Markov models and selected applications in speech recognition**. *Proceedings of the IEEE* 1989, **77**(2):257–286.
4. Bilmes JA: **A gentle tutorial of the EM algorithm and its application to parameter estimation for gaussian mixture and hidden Markov models** 1998. [Www.icsi.berkeley.edu/techreports/1997/html].
5. Durbin RD: *Biological sequence analysis—probabilistic models of proteins and nucleic acids.* Cambridge: Cambridge University Press 1998.
6. Murphy KP: **Dynamic Bayesian Networks: Representation, Inference and Learning— Chapter 3. Exact inference in DBNs** 2002, :46–48. [Www.ai.mit.edu/murphyk/Thesis/thesis.html].
7. Newton R: **Detecting deletions in bacterial microarray data** 2003. [MSc Thesis—Birkbeck College, University of London].
8. Newton R, Hinds J, Wernisch L: **A Hidden Markov Model web application for analysing bacterial genomotyping DNA microarray experiments**. *Applied Bioinformatics* 2006, **5**:211–218.
9. Viterbi AJ: **Error bounds for convolutional codes and an asymptotically optimum decoding algorithm**. *IEEE Transactions on Information Theory* 1967, **13**:260–269.
